# Exploring clinician interpretability of AI decision support systems for surgical anatomy recognition

**DOI:** 10.1038/s41598-026-74953-y

**Published:** 2026-10-24

**Authors:** Danyal Zaman Khan, Tobias Joseph Adams, Anjana Wijekoon, Roxana Ramirez Herrera, Sophia Bano, Peter McCulloch, Danail Stoyanov, Matthew J Clarkson, Enrico Costanza, Ann Blandford, Hani Joseph Marcus, CARES Evaluation Group, Mohammed Mustafa Adawi, Joao Paulo Almeida, Jensen Ang, Anouk Borg, Roman Bošnjak, Alexandros Boukas, Aswin Chari, Lekhaj C Daggubati, Neil L Dorward, Samuel Emerson, Laurence Johann Glancz, Paul Gwammache, John Gerrard Hanrahan, Joachim Starup-Hansen, Lauren Harris, Mark Hughes, Abhiney Jain, Adham M Khalafallah, Taufiq Khan, Muhammad Sohaib Khan, Ramez Wadie Kirollos, Angelos G Kolias, Jacek Kunicki, Ruth Lau, Juan Carlos Fernandez-Miranda, Ramesh Nair, Nicola Newall, Amr Nimer, Fabio Massimo Oddi, Piero Andrea Oppido, Piotr Paździora, Hafiz Muhammad Irfan Razzaq, Luis Alejandro Flores Salazar, Olabisi Sanusi, Thomas Santarius, Sebastian Senger, Jonathan Shapey, Nathan A Shlobin, Nicholas Wake Macmeikan Thomas, Sebastian Miguel Toescu, Georgios Tsermoulas, Juan Francisco Villalonga, Adam Williams, Nina Yoh, Brett E Youngerman, Gabriel Zada

**Affiliations:** 1 Dept of Translational Neuroscience and Stroke, UCL Queen Square Institute of Neurology, University College London, London, UK; 2 Dept of Neurosurgery, National Hospital for Neurology & Neurosurgery, Queen Square, London, UK; 3 Manchester University NHS Foundation Trust, Manchester, UK; 4 Department of Computer Science and UCL Hawkes Institute, University College London, London, UK; 5 UCL Human Computer Interaction Centre, University College London, London, UK; 6 Nuffield Department of Surgical Sciences, University of Oxford, Oxford, UK; 7 Al-Zaitoun Specialized Hospital, Cairo, Egypt; 8 Indiana University, Bloomington, USA; 9 National Neuroscience Institute, Singapore, Singapore; 10 Oxford University Hospitals NHS Foundation Trust, Oxford, UK; 11 Institute of Child Health, University College London, London, UK; 12 Washington Brain & Spine Institute, Rockville, USA; 13 University of Washington, Seattle, USA; 14 Queen’s Medical Centre Nottingham, Nottingham, UK; 15 Harvard University, Cambridge, USA; 16 Department of Clinical Neurosciences, NHS Lothian, Edinburgh, UK; 17 Neurosurgery Department, University of Miami, Miami, USA; 18 Queen Elizabeth University Hospital, Birmingham, UK; 19 Lady Reading Hospital Peshawar, Peshawar, Pakistan; 20 University of Cambridge, Cambridge, UK; 21 National Research Institute of Oncology, Warsaw, Poland; 22 Joan XXIII University Hospital, Tarragona, Spain; 23 Stanford University, Stanford, USA; 24 Charing Cross Hospital, London, UK; 25 Imperial College London, London, UK; 26 University of Rome “Tor Vergata”, Rome, Italy; 27 Sapienza University of Rome, Rome, Italy; 28 Department of Neurosurgery, Medical University of Silesia in Katowice, Katowice, Poland; 29 Punjab Institute of Neurosciences, Lahore, Pakistan; 30 University Medical Centre Ljubljana, Ljubljana, Slovenia; 31 Hospital Barros Luco Trudeau, Santiago, Chile; 32 Oregon Health & Science University, Portland, USA; 33 Cambridge University Hospitals, Cambridge, UK; 34 Department of Neurosurgery, Sozialstiftung Bamberg, Bamberg, Germany; 35 King’s College London, London, UK; 36 Columbia University Irving Medical Center, New York, USA; 37 Queen Elizabeth Hospital in Birmingham, Birmingham, UK; 38 LINT, Facultad de Medicina, Universidad Nacional deTucumán, San Miguel de Tucumán, Argentina; 39 North Bristol NHS Trust, Bristol, UK; 40 University of Southern California, Los Angeles, USA

**Keywords:** Artificial intelligence, Computer vision, Decision support systems, Surgery, Computational biology and bioinformatics, Health care, Mathematics and computing, Medical research

## Abstract

Artificial intelligence (AI) decision support systems for surgery hold promise but face barriers to adoption, particularly around the interpretability of their outputs. We conducted an international cross-sectional survey of 47 neurosurgeons to evaluate perspectives on literature-derived explanatory techniques for AI-generated anatomical segmentations, using endoscopic pituitary surgery as a high-risk exemplar. Participants ranked certainty scores, certainty maps, saliency maps, scene similarity scores, and nearest-neighbour illustrations, and rated them using a modified Explanation Satisfaction Scale alongside free-text feedback. Certainty-based techniques were consistently ranked and rated highest - valued for aligning with surgical decision-making by conveying confidence (via scores) and anatomical boundaries (via maps). Saliency- and similarity-based methods were judged less clinically relevant and better suited to educational settings. Therefore, certainty-based techniques appear most acceptable to surgeons for improving the interpretability of AI-assisted anatomical navigation systems, though their impact on trust calibration and performance requires prospective evaluation across surgical domains.

**Supplementary Information:**

The online version contains supplementary material available at https://doi.org/10.1038/s41598-026-74953-y.

## Introduction

Artificial intelligence (AI) tools for surgical applications have demonstrated relatively slow uptake compared to other medical fields, particularly in decision support, despite their theoretical promise for improving safety, effectiveness and efficiency^1–3^. This is in part due to the complexity of deploying AI systems in the operating room environment. In order to analyse surgical procedures in detail, surgical computer vision (SCV), a rapidly expanding subtype of AI that analyses surgical video data, must be optimised for accurate real-time performance^4^. This is technologically challenging as surgical video data is high-dimensional, temporal, non-stationary with rapid viewpoint shifts, and often subject to occlusions and variable lighting^1,2^. From a human factors perspective, surgery is already a high-stakes, cognitively demanding and dynamic environment, and so any technology introduced must be carefully integrated into the surgical workflow^5^.

Surgical anatomy recognition is a fundamental process in any surgical procedure, and has been targeted as a task which could be improved by AI assistance, synergising with existing tools that surgeons use for orientation and navigation (e.g. image-guidance)^6–9^. This leverages SCV technology, pioneered in histology and radiology for pathology detection, which has already been translated successfully into practice in those clinical domains^10^. Numerous AI clinical decision support systems (AI-CDSS) to assist surgical anatomy recognition by segmenting key structures have been developed recently for a variety of surgeries, for example, laparoscopic cholecystectomy and endoscopic pituitary surgery, and have been shown to improve surgeon anatomy recognition and thus surgical safety^4,9,11,12^. Many factors, such as interpretability, affect the integration of AI-CDSS output by clinicians into their decision-making^4,12,15^. The taxonomy of interpretability and related concepts (e.g. explainability, transparency, cognitive integration) is complex and varies depending on the perspective (AI models, clinical research, human factors, cognitive neuroscience) and resultantly is poorly defined in literature^13–15^. For example, interpretability from a machine learning perspective has a different meaning to its use in human factors or lay domains.

For the purposes of translating AI-CDSS, we focussed on exploring clinician interpretability, that is, techniques which improve a clinician’s understanding of the quality or mechanism of an AI-CDSS output, such that the output can be integrated (or not) into decision making. Interpretable AI recommendations should therefore facilitate clinician acceptability, usability, appropriate calibration of trust and reliance, and thus optimise AI-CDSS effectiveness and safety^16^. The interpretability of AI-CDSS outputs is likely to be influenced by AI model factors, AI-human interface design factors and human user factors (e.g. digital literacy). Modifying these factors may allow improvement of interpretability and subsequent safe uptake of AI-CDSS^14, 17^. In the related and more established field of computer vision for medical imaging, various techniques that provide additional information alongside the primary model output have shown promise in improving perceived interpretability and acceptability of AI tools to clinicians^18^. The most common techniques to improve interpretability include attribution-based (e.g. saliency maps) and example-based methods, whilst uncertainty-based methods are uncommon and potentially overlooked^13, 18^. Many of these techniques can be presented in many different ways, such as text, numeric, visual and visuospatial formats^19^. Ultimately, these techniques should be evaluated from both the system designer’s perspective and the user’s perspective, and when deployed intra-operatively in real-time must present a careful balance of information at the right time^4, 15, 20^.

The influence of techniques to improve surgical AI-CDSS interpretability, decision making and clinical impact is unknown. Its importance is underlined by recommendations from international guidelines in the clinical evaluation of AI systems, such as DECIDE-AI^21^. We sought to explore surgeons’ perspectives on various literature-derived AI-CDSS interpretability improving techniques. We used endoscopic pituitary tumour surgery as an exemplar, where SCV-based AI-CDSS for anatomy recognition has already been developed, and where this anatomy recognition is amongst the highest risk of all surgeries owing to the tumour’s close proximity to life-sustaining neurovascular structures^9^.

## Methods

### Aim

The primary aim of this study was to evaluate different interpretability improving techniques, displayed alongside AI-generated anatomical segmentation outputs, in endoscopic pituitary surgery. This evaluation is from the perspective of the end-user (surgeon) and will inform the design of future SCV tools to enhance intraoperative decision-making and surgical education.

### Study design and participants

A cross-sectional survey-based study administered electronically via the Qualtrics XM software program (Qualtrics, Seattle, WA) was adopted. Eligible respondents included neurosurgeons and neurosurgical trainees with a minimum of one year’s experience in performing the endoscopic transsphenoidal pituitary surgical approach. The study was advertised through national neurosurgical organisations (predominantly UK-based), trainee networks (predominantly UK-based), and social media channels (internationally, via X and LinkedIn).

### Ethical considerations

All methods were carried out in accordance with the principles of the Declaration of Helsinki and were approved by the University College London Ethics Committee (UCLIC-2024_009). Participation was voluntary, and informed consent was obtained from all participants. Participants were offered collaborative authorship as part of the “CARES Evaluation Group” to recognise their contribution.

### Survey content and structure

Baseline characteristics were collected – including demographics (age, biological sex); clinical experience in performing endoscopic transsphenoidal pituitary surgery; and frequency of respondent interaction with AI tools in their clinical or academic practice (Supplementary Information 1).

**Fig. 1:**
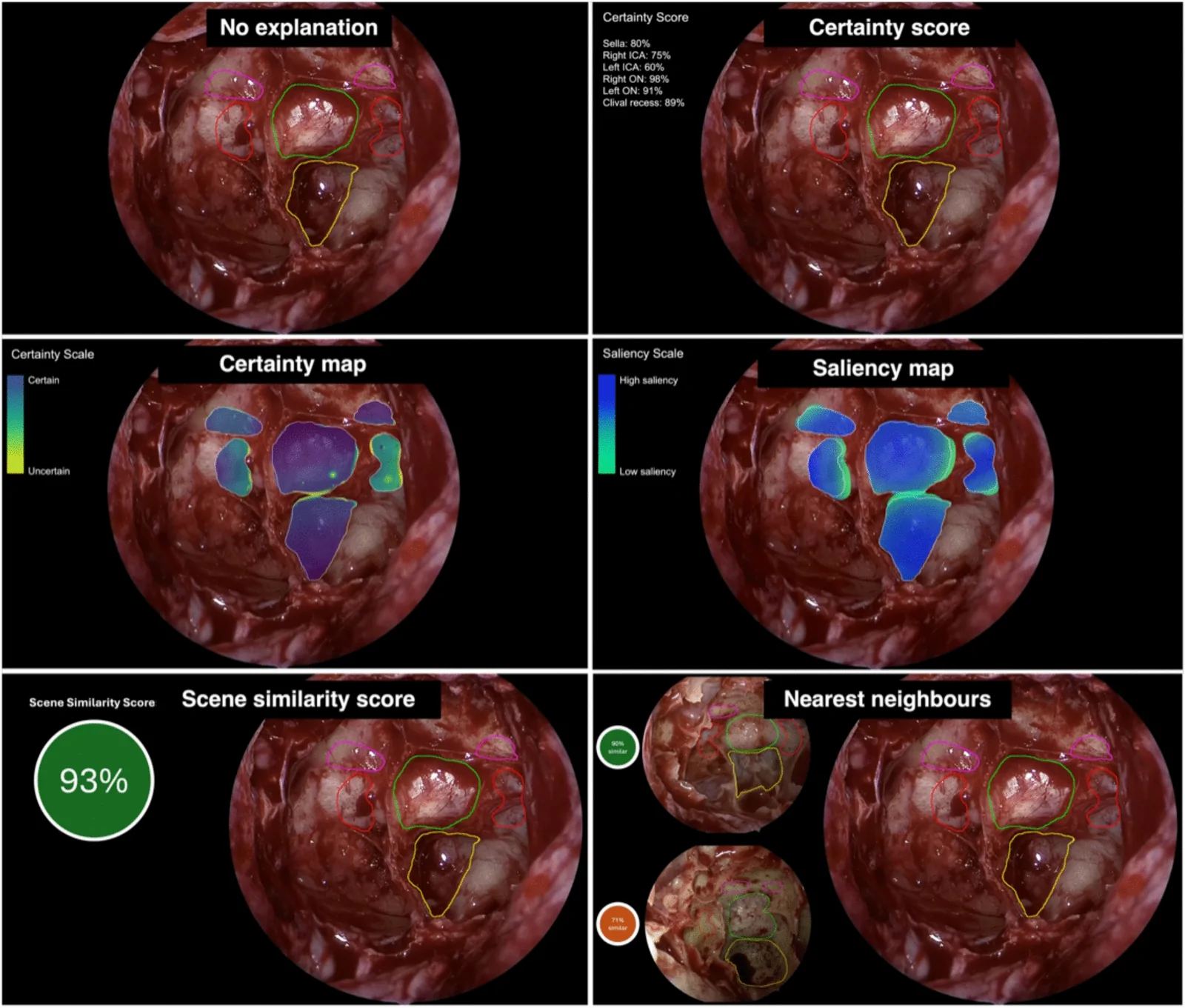
Overview of explanatory techniques presented to surgeons during the study. The Certainty Score was described as a quantification of the AI model’s certainty in the segmented area of each anatomical structure, which would represent the average pixel-wise certainty across the entire structure as a percentage^23^. The Certainty Map was described as a display of the AI model’s certainty in identifying the area for each anatomical structure, with dark blue indicating strong certainty, while yellow-green indicated uncertainty^24^. The Saliency map was described as the regions in the image that the AI model considered most important for identifying anatomical structure, with dark blue indicating high saliency (where the model focuses most of its attention), while green-turquoise represented lower saliency^25, 26^. The Scene Similarity Score was described as a numeric representation of how closely the current surgical scene matched the examples the AI model was trained on, with a higher percentage suggesting the model is working within its “comfort zone,” making it more likely that its predictions are accurate^27,28^. The Nearest Neighbours technique was described as an illustration of how similar the current image is to examples the AI was trained on and provides two “nearest neighbour” images for comparison, along with their scene similarity scores relative to the primary surgical view^27,28^. No explanation means the AI overlay alone without any interpretability improving techniques.

Participants then received a brief explanation of the underlying AI model featured in subsequent sections, representing the minimum level of information necessary to understand the tool’s function. Details of this tool have been described elsewhere^9^. Participants were then shown the same still frame from the sellar phase of pituitary surgery with an illustrative AI overlay incorporating one of five different interpretability improving techniques: certainty scores, certainty maps, saliency maps, scene similarity scores and nearest neighbours illustration (Fig. 1). The design of the primary model output (i.e. segmentation outline overlay) which the interpretability improving techniques were presented alongside was derived from a prior study of surgeon preferences^22^. The order in which techniques were presented was randomised for each participant.

Our group’s choice of interpretability improving techniques was informed by the wider CV literature, based on their perceived translation potential for real-time (intra-operative) and offline (peri-operative) surgical applications. The chosen techniques were: saliency maps (a type of attribution-based technique, the most prevalent in the literature), nearest neighbour (a type of example-based technique, the second most prevalent in the literature) and certainty estimation (often described as overlooked in CV, but common in wider predictive tool literature)^14,18, 23–28^. Each technique was displayed in numeric or text and visual or visuo-spatial formats, except for saliency maps which are inherently visuo-spatial only. As the focus of the study was to assess user perspectives on each technique conceptually, the displays were illustrative rather than computed, with explanatory text about each technique’s intended function and potential interpretation (Fig. 1). Numeric values for certainty estimation, scene similarity and nearest neighbour techniques were illustrated using Microsoft PowerPoint (Microsoft Corporation, Redmond, WA, USA). Colour overlays for certainty and saliency map techniques were illustrated using Photopea illustrator (www.photopea.com). Values and colour-coding illustrated were hypothetical and selected to be plausible for the anatomical segmentation shown.

After reviewing each technique individually, participants completed a modified Explanation Satisfaction Scale (ESS) to provide contextualised Likert-based ratings of explanatory technique understandability, satisfaction, detail sufficiency, usefulness and accuracy^20^. This scale was modified to remove two redundant and inapplicable question items. This was accompanied by an open question on each technique’s utility for clinical or educational applications, and wider general feedback. Finally, participants were presented all five explanatory techniques together, along with just the AI overlay (without interpretability improving techniques), and asked to rank by preference.

### Data analysis

Descriptive summary statistics were produced for baseline characteristics, ranking and ESS scores. Internal consistency of the modified ESS was assessed using Cronbach’s α, computed across the five retained items separately for each technique, to justify aggregation of items into a mean score. For ranking data, a weighted average ranking was calculated by dividing the weighted sum by the total number of rankings per explanatory technique. The weighted sum for each technique was calculated as the sum of the rank frequency at each position (1–6), multiplied by the corresponding rank position value (1st = 1, 2nd = 2, …). Free-text responses were thematically analysed using an inductive approach to identify the 3 most prominent feedback domains per explanatory technique. Responses were typically brief, descriptive and technical in nature, describing specific perceived advantages or disadvantages of each display rather than complex experiential accounts, and therefore afforded limited scope for interpretative divergence, so coding by a single coder was considered sufficient (DZK). Representative quotations were selected to illustrate the range of opinion within each domain, including dissenting views.

Comparative statistics were performed using Excel (V16.1, Microsoft, USA) and R (V4.4.3, R Studio). Differences in technique rankings and ESS scores were assessed using the non-parametric Friedman test for repeated measures. For each analysis, the Friedman χ^2^ statistic, degrees of freedom (DoF = k–1; DoF = 4 for the five-technique ESS comparison and DoF = 5 for the six-option ranking comparison), effective sample size, Kendall’s W, and p-values were reported. Effect size for pairwise comparisons was quantified using Cohen’s r (z divided by the square root of the number of pairs; with small ≈ 0.1, medium ≈ 0.3, and large ≈ 0.5). P-values from post-hoc pairwise Wilcoxon signed-rank tests were adjusted for multiple comparisons using the Holm procedure. Spearman’s correlation coefficient was calculated to assess the relationship between mean ESS scores and weighted average rank to assess the consistency of surgeon feedback.

## Results

### Sample characteristics

An international cohort of 47 neurosurgeons completed the study survey (Table 1). The majority of participants practised in the UK (n = 22, 46.8%) or USA (n = 11, 23.4%). Other countries of practice represented were: Italy (n = 2, 4.3%), Pakistan (n = 2, 4.3%), Singapore (n = 2, 4.3%), Poland (n = 2, 4.3%), Egypt (n = 1, 2.1%), Chile (n = 1, 2.1%), Germany (n = 1, 2.1%), Argentina (n = 1, 2.1%), Slovenia (n = 1, 2.1%) and Spain (n = 1, 2.1%). The cohort was diverse in terms of age, surgical experience and experience with AI-assisted technologies (Table 1). Of note, most participants were attending level (68.1%), and the majority used AI for clinical or academic tasks at least once a month (70.2%).

**Table 1:** Participant demographics and AI experience.

Characteristic	Category	Count (N = 47)	Percentage (%)
Age Distribution	25–34 years old	11	23.4
	35–44 years old	24	51.1
	45–54 years old	5	10.6
	55–64 years old	5	10.6
	65 + years old	2	4.3
Biological Sex	Male	40	85.1
	Female	6	12.8
	Prefer not to say	1	2.1
Professional Role	Neurosurgery Consultant or Attending	32	68.1
	Neurosurgery Registrar or Resident	13	27.7
	Neurosurgery Senior Fellow (post-training)	2	4.3
Years of Experience Performing Endoscopic Pituitary Surgery	1 to 5 years	20	42.6
	5 to 10 years	15	31.9
	10 to 20 years	8	17.0
	Over 20 years	4	8.5
AI Usage Areas†	Academic research	28	31.1
	Administrative tasks	18	20.0
	Clinical decision-making	12	13.3
	Disease diagnosis	6	6.7
	Medical imaging interpretation	7	7.8
	Robot-assisted surgery	6	6.7
	Teaching	1	1.1
	None	12	13.3
AI Interaction Frequency	Every day	11	23.4
	Every week	14	29.8
	At least once per month	8	17.0
	At least once per year	4	8.5
	Never	10	21.3

^†^Participants could select multiple options; therefore, percentages are calculated based on the total number of selections.

### Comparative analysis of explanatory techniques

**Fig. 2:**
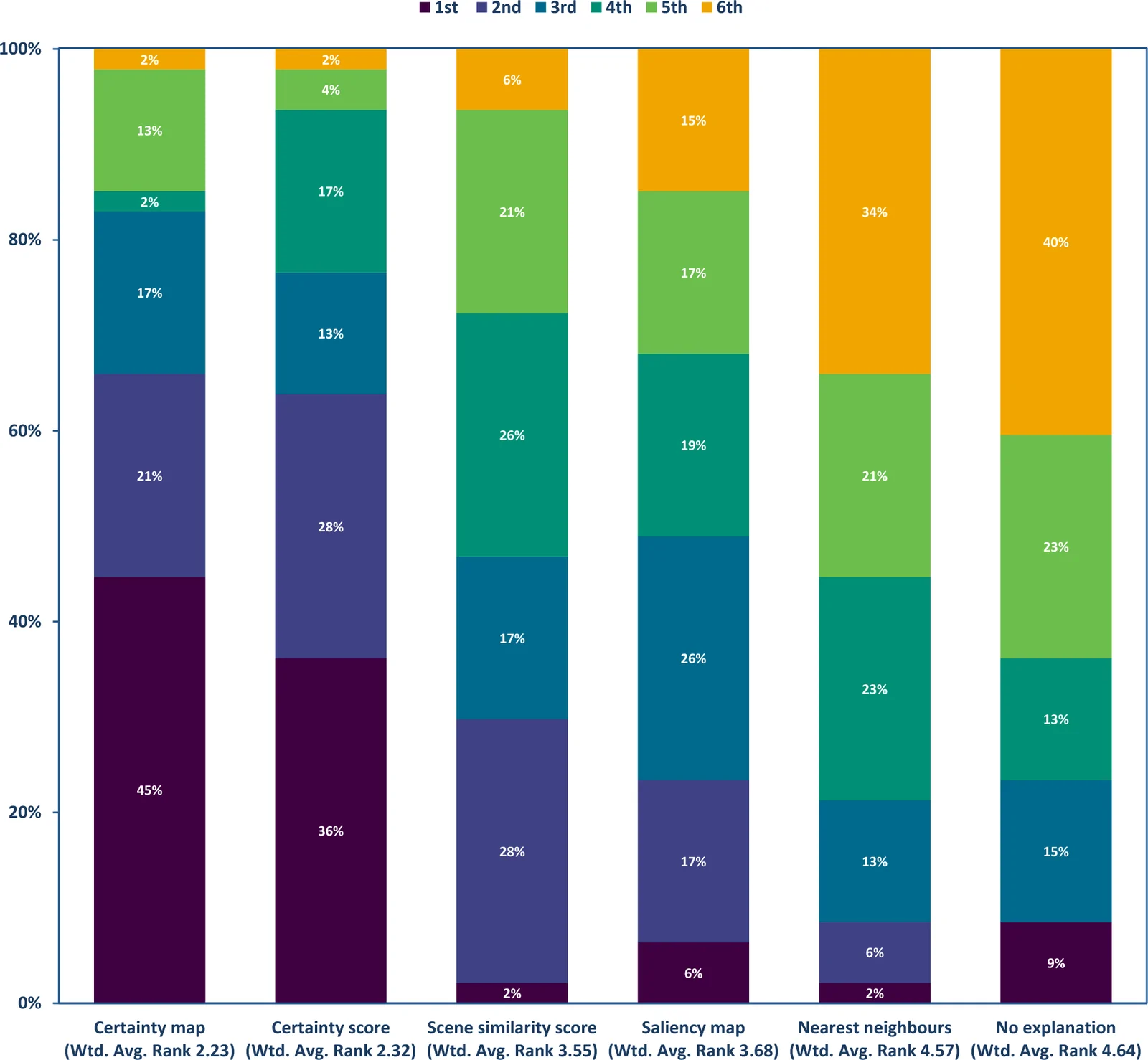
Stacked bar chart display of ranking frequency of each technique. Wtd. Avg. Rank = Weighted Average Rank. No explanation = AI overlay alone without any interpretability improving techniques.

Certainty-based explanatory techniques - spatial maps and numeric per-structure summary scores - emerged as the top-ranked and top ESS scored options by surgeons (Tables 2, 3, 4, Fig. 2).

**Table 2:** Summary of modified Explanation Satisfaction Score results for each technique.

Technique	Certainty map	Certainty score	Scene similarity score	Nearest neighbours	Saliency map
This explanatory technique is **satisfying**	4.32	4.13	3.96	3.70	3.91
This explanatory technique shows me how **accurate** the AI prediction is	4.32	4.38	4.11	3.64	3.53
This explanatory technique has sufficient **detail**	4.30	4.19	3.51	3.74	3.64
Using this explanatory technique, I **understand** how the AI works	4.00	3.85	3.70	3.83	3.62
This explanatory technique is **useful** to my surgical or educational goals	3.96	3.85	3.70	3.49	3.38
Mean Score	4.18	4.08	3.80	3.68	3.62

**Table 3:** Pairwise Wilcoxon Signed-Rank Test Results for ESS Scores, with p-values and corresponding effect size via Cohen’s r.

Technique	Certainty map	Certainty score	Scene similarity score	Nearest neighbours	Saliency map
Certainty map	—	—	—	—	—
Certainty score	0.503 (r = 0.22)	—	—	—	—
Scene similarity score	0.038* (r = 0.40)	0.049* (r = 0.38)	—	—	—
Nearest neighbours	0.006* (r = 0.49)	0.006* (r = 0.49)	0.567 (r = 0.16)	—	—
Saliency map	< 0.001* (r = 0.64)	0.002* (r = 0.54)	0.503 (r = 0.22)	0.617 (r = 0.07)	—

Significant comparisons (p < 0.05) are marked with an asterisk (*). Diagonal cells (—) indicate comparisons of a technique with itself. ESS = explanation satisfaction scale.

**Table 4:** Pairwise Wilcoxon Signed-Rank Test Results for Rankings, with p-values and corresponding effect size via Cohen’s r.

Technique	Certainty map	Certainty score	Scene similarity score	Nearest neighbours	Saliency map
Certainty map	—	—	—	—	—
Certainty score	1.000 (r = 0.04)	—	—	—	—
Scene similarity score	0.006* (r = 0.48)	0.003* (r = 0.52)	—	—	—
Nearest neighbours	< 0.001* (r = 0.68)	< 0.001* (r = 0.77)	0.006* (r = 0.48)	—	—
Saliency map	< 0.001* (r = 0.64)	0.005* (r = 0.50)	1.000 (r = 0.04)	0.069 (r = 0.35)	—
AI overlay alone without interpretability improving technique	< 0.001* (r = 0.73)	< 0.001* (r = 0.73)	0.006* (r = 0.49)	1.000 (r = 0.05)	0.071 (r = 0.34)

Significant comparisons (p < 0.05) are marked with an asterisk (*). Diagonal cells (—) indicate comparisons of a technique with itself.

Firstly, there was a significant difference in ESS scores between techniques (Friedman test: χ^2^ = 32.1, DOF = 4, N = 47, p < 0.001, W = 0.17). Internal consistency of the ESS was high for every technique (Cronbach’s α 0.88–0.92), supporting aggregation of the five items into a mean score. There were no missing ESS or ranking responses. Certainty map and certainty score were rated significantly higher than saliency maps, scene similarity scores, and nearest neighbours on post-hoc pairwise Wilcoxon signed-rank tests (Table 3). There was no significant difference between the certainty map and certainty score (Table 3; p = 0.50, r = 0.22).

Regarding ranking, there was a significant difference in rankings between techniques (Friedman test: χ^2^ = 73.6, DOF = 5, N = 47, p < 0.001, W = 0.31). Certainty map and certainty score were ranked significantly higher than saliency maps, scene similarity scores, nearest neighbours and AI overlay alone on post-hoc pairwise Wilcoxon signed-rank tests (Table 4, Supplementary Information 2). Again, there was no significant difference between certainty map and certainty score (Table 4; p = 1.0, r = 0.04). Neither the saliency map nor the nearest-neighbour illustration was ranked significantly higher than the AI overlay alone without any interpretability improving techniques (p = 0.071 and p = 1.000 respectively).

 Mean ESS Scores and the weighted average ranks were negatively correlated across the five techniques (Spearman's correlation coefficient -0.90; p = 0.083), indicating close concordance between ratings and rankings.

### Certainty map: quantitative & qualitative feedback

**Fig. 3:**
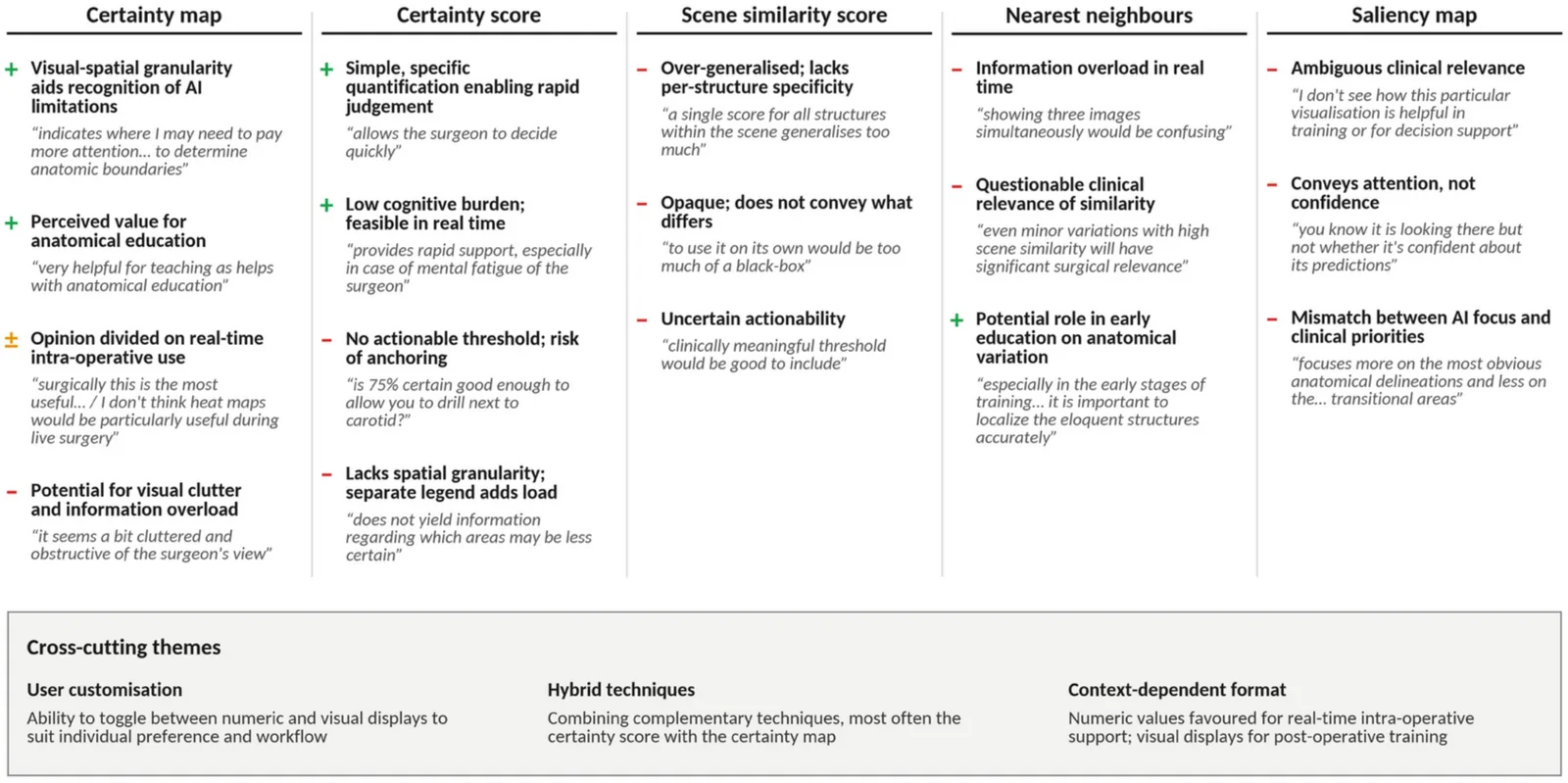
Visual summary of thematic analysis of qualitative data per technique. +  = facilitator,— = barrier, ±  = divided opinion.

The certainty map explanatory technique had the highest average rank, the highest mean ESS score and the highest mean score in all domains except in the conveying accuracy domain (Table 2, Fig. 2). As a spatial display of the certainty score, it appeared to improve granularity, comprehension and perceived utility, but sacrificed simplicity in conveying AI accuracy (Tables 2 & 3). This was reflected in the thematic analysis (Fig. 3) which highlighted the *surgical utility of visual-spatial feedback* for these complex 3D anatomical structures - particularly with respect to recognizing the limitations of the AI overlays (e.g., “indicates where I may need to pay more attention… to determine anatomic boundaries”, “which areas may require further adjuncts”, and “areas of uncertainty are helpful additions, as they could help guide decision making, and inform the degree to which the AI should be trusted”). This translated into *the potential for intuitive improvements to training and surgical performance *- this was particularly the case for offline educational applications (“very helpful for teaching as helps with anatomical education”). However, its application in real time during surgery split opinions - with some surgeons for (e.g. “surgically this is the most useful…especially the medial border of the ICA, where certainty happens to be low so great caution needed in following this too closely”) and some against (“I am not sure about its utility for decision support” and “I don’t think heat maps would be particularly useful during live surgery”). This is likely in part due to the overlay design and its *potential for information overload*, especially in real time, with this technique (“it seems a bit cluttered and obstructive of the surgeon’s view” and “too busy for my taste”).

### Certainty score: quantitative & qualitative feedback

The certainty score, presented on a per-structure basis (Fig. 1), had the second-highest average rank and mean ESS score (Table 2, Fig. 2). It had the highest score in the conveying accuracy ESS subdomain - reflecting its simplicity and specificity as an explanatory technique. Upon analysis of the qualitative feedback on the clinical utility of certainty scores , key themes revolved around the pros and cons of a numeric value. Many respondents appreciated the *value of simplistic quantification*, allowing “the surgeon to decide quickly” using “concrete” information which would be “neat to display in real-time”, whilst potentially reducing cognitive overload (“provides rapid support, especially in case of mental fatigue of the surgeon”). However, *uncertainty in utility for real-world decision-making* was expressed, emphasising the need for better definitions of what numeric value is meaningful (“what cut off is used for clinical decision making is uncertain - is 75% certain good enough to allow you to drill next to carotid?” and “it might be prone to an anchoring effect…I might think 80 is ok if other scores are in the 60s, or I might think it’s unacceptable if all the others are in the 90s”). Furthermore, *lack of granularity and nuance* emerged as a recurring concern, particularly for users who voluntarily described themselves as “visual learners”. Specifically, the lack of spatial information was cited repeatedly (“does not yield information regarding which areas may be less certain”). Furthermore, some users felt numeric displays were more cognitively demanding, especially if displayed separately (“may slow down application of AI in a surgical setting as based on reading numbers…easier to rely on colour maps” and “prefer more visual indicators vs numbers as may be a bit more challenging to interpret fully intra-op if lots of info on screen”). This issue seemed to in part be due to the certainty score’s numeric nature but also in its presentation, separate from the overlays in a legend (“difficult to look away from the camera to read the numbers” and “Separating data presentation increases cognitive load… I find it strenuous to interpret the confidence of annotations with this format”).

### Scene similarity score: quantitative & qualitative feedback

After the certainty-based techniques (map and score), the scene similarity score technique had the next highest average rank and mean ESS score (Table 2, Fig. 2). On thematic analysis, surgeons found it *over generalized and lacking specificity* (e.g., "a single score for all structures within the scene generalizes too much") and *challenging to interpret* (e.g., “To use it on its own would be too much of a black-box”, “does not explain what was similar/different between this image and the examples that the model was trained”, and “what the model perceives to be similar is NOT necessarily actually similar, this does not increase my confidence in the classification at all”). Like with the certainty score, the *uncertain actionability* was expressed (e.g., “clinically meaningful threshold would be good to include” and “% for each anatomical structure would be more helpful”).

### Nearest neighbours: quantitative & qualitative feedback

This technique ranked last in average rank and second last in mean ESS scores (Table 2, Fig. 2). By leveraging examples of similar cases, it performed relatively well in conveying an understanding of how the AI worked (Table 2). Core themes emerging from surgeon feedback on its utility were concerns about *information overload potential* (e.g. “too confusing”, “too much information in real-time” and “showing three images simultaneously would be confusing”) and the *questionable clinical relevance of similarity* (“even minor variations with high scene similarity will have significant surgical relevance”). Nevertheless, there was an acknowledgement of its potential role for *early education of anatomical variations* aimed at less experienced members of the clinical team.

### Saliency map: quantitative & qualitative feedback

Saliency Map was the least effective technique overall according to the mean ESS score, and ranked second last in rankings (Table 2, Fig. 2). It scored particularly poorly on its usefulness to users’ goals (Table 2), suggesting that while visually engaging, simply highlighting where an AI is "looking" is insufficient for user satisfaction and trust. Thematic analysis reflected this *ambiguous clinical relevance* (e.g., “whilst this helps understand how the AI works and generates its suggestions, I don’t see how this particular visualisation is helpful in training or for decision support” and “you know it is looking there but not whether it’s confident about its predictions”). Furthermore, when compared to certainty maps, surgeons felt saliency maps demonstrated a *mismatch between AI focus and clinical priorities* (e.g. “focuses more on the most obvious anatomical delineations and less on the neighbouring or transitional areas, which are the most dangerous from a surgical point of view”).

### Wider feedback

Thematic analysis of optional wider feedback distilled 3 key themes. Firstly, an emphasis on *user customisation* in designing such AI systems to allow for differences in preference and clinical workflow - specifically, the option to toggle between numeric and visual displays (e.g. the certainty score and certainty map). Secondly, consideration for *hybrid techniques* was requested to synergise the benefits: for example, combining the certainty score and certainty map techniques. Finally, numeric values were considered more feasible for use as real-time intra-operative decision support, whilst visual displays were felt to be particularly useful for post-operative training and education.

## Discussion

### Principal findings

This study provides novel insights into surgeon perspectives on the design of user-centred AI-CDSS for anatomical navigation. Using an existing anatomy recognition assistance computer vision AI model developed for endoscopic pituitary surgery as an exemplar, we explored surgeon perceptions of a variety of literature-derived techniques which seek to make the decisions of otherwise “black-box” AI systems more interpretable, which in turn may impact trust, safety and translational impact. To our knowledge, this is the first study of its kind in surgical computer vision, addressing the unique challenges surgery presents when compared to imaging, histopathology or diagnostic endoscopy computer vision applications.

We found that metrics that are based on the concept of certainty, that is, how certain an AI model was in its predictions, aligned best with surgeons’ perspectives and cognitive workflow. Quantifying uncertainty is a core tenet of clinical decision-making, particularly in the context of using decision support systems, as it helps clinicians critically appraise AI assistants and judge their reliability and trustworthiness^29–32^. The Certainty Map, a visual-spatial representation of this, received the highest overall ESS score and was the most frequently top-ranked technique. This preference was driven by its perceived utility in providing intuitive feedback on anatomical boundaries between structures and areas of uncertainty where additional adjuncts may need to be utilised (e.g., Doppler ultrasound or image-guidance). The Certainty Score, which provides a summary numeric value for each identified anatomical structure, also performed well, scoring highest in the "conveying accuracy" ESS domain. Its simplicity was seen as a key advantage for rapid interpretation, particularly in a cognitively demanding intra-operative environment. Hybrid numeric and visual designs or the ability to toggle between them were endorsed by multiple participants.

Conversely, techniques that focused on the internal workings of the AI without direct clinical relevance - namely the Saliency map, Scene similarity score, and Nearest neighbours - were ranked significantly lower. Of these, scene similarity score performed best in the conveying accuracy ESS subdomain, potentially reflecting its interpretation simplicity as a numeric value, akin to the certainty score. These techniques were suggested to have greater potential for educational purposes, particularly for junior trainees learning to identify anatomical variations, but were deemed unsuitable for real-time intraoperative use. Practically, out-of-distribution data and/or domain shift (i.e. the operative scene analysed is significantly different to the data the AI model was trained on) can be inferred from some certainty estimates, and so scene similarity and nearest neighbour type displays may be partially redundant in practice^32^.

### Findings in the context of the literature

Our findings resonate with a growing body of literature on the critical role of techniques to improve AI interpretability in bridging the translational gap of surgical AI systems. This is particularly relevant for deep learning methods, which are inherently “black box” in terms of their decision-making processes, making the integration of their outputs by human users challenging and interesting^33^. The terminology used to study this encompasses related concepts such as explainability and transparency, but is used very heterogeneously in the literature, with different meanings across different scientific disciplines, and needs alignment in the future.

The preference for certainty-based explanations, such as certainty maps and scores, aligns with recent work highlighting that transparent communication of an AI model’s limitations is a cornerstone of responsible clinical deployment^34^. A recent review on explainable AI for medical imaging suggested that providing uncertainty estimations can improve the trust calibration of clinicians, preventing both over- and under-reliance^30^. The Certainty map, in particular, by translating abstract uncertainty into an intuitive visual format, provides a level of comprehension that may surpass simple numeric metrics, reflecting its high ranking and ESS scores in our study. However, there is likely a cognitive cost to this spatial information, particularly for real-time applications. , On the other hand, some studies suggest that oversimplification of AI output can lead to an “anchoring effect” where clinicians may inappropriately trust a single high-certainty number without understanding the underlying spatial or contextual nuances - a concern explicitly raised by participants in our study^32, 35^. Possible strategies to mitigate anchoring bias further could include encouraging users to make initial decisions themselves before utilising AI assessment, which could be enabled in this context by allowing users to toggle the AI overlay on or off^35^. 

Whilst conceptually appealing, these methods need further study for safe integration into clinical decision-making. Priorities include developing certainty thresholds that indicate acceptable reliability; monitoring whether certainty metrics remain well calibrated to model accuracy (to guard against confidently incorrect predictions); and exploring whether their use improves surgeon acceptability, performance and trust calibration^4, 30, 32^. A related consideration is what kind of uncertainty a given metric actually conveys. For example, per-structure certainty numeric scores calculated by aggregating model prediction probabilities have been deployed intra-operatively in real time^36,37^. These indicate how ambiguous the model finds the scene, not how familiar the scene is to it, because the model’s parameters are fixed at inference^36, 37^. Methods that assess familiarity explicitly, including Bayesian and ensemble-based approaches, often require multiple forward passes per frame and so multiply computational cost relative to a single deterministic pass. This computational cost currently constrains intra-operative use, but not applications where inference latency is not limiting, such as post-operative review^32^.

The challenges observed with saliency maps are also well-documented. While a popular technique in technical AI literature, saliency has been criticised in medical contexts for not providing a sufficient causal link between the model’s focus and the clinical task at hand^38^. Clinicians need to know not just *where* the model is looking, but *why* those areas are important and what the confidence level is for the resulting prediction. This study confirms that for a high-stakes task like identifying critical neurovascular structures, an explanation that lacks this crucial contextual and confidence information is of limited value.

### Strengths and limitations

This international study was built on a foundation of existing evidence - an AI system with demonstrated surgeon benefit for use as an exemplar case study and AI overlay designs that had already been optimised - allowing the study to focus specifically on AI system interpretability considerations^9, 22^. Other strengths include the combination of validated quantitative and insightful qualitative feedback attained, which may be relevant to other surgical computer vision applications.

Limitations include the UK- and US-centric sample recruited despite best efforts to disseminate the opportunity widely - likely reflecting the natural networks of the author group - and reducing its representativeness of the global surgical community. Furthermore, respondents may have rated techniques as satisfying or dissatisfying when in fact their understanding of the process being explained was piecemeal or flawed^20^. Additionally, the analysis was performed on still, anonymised images to reflect the breadth of potential SCV applications, including for example, post-operative coaching using key images^39^. As only a single surgical image was used for clinical context, the consistency of these preferences when applied in different intra-operative environments with the dynamic challenges of live surgery (e.g., anatomical variation, rapid viewpoint shifts and increased cognitive load) needs further study. , this study primarily explored interface display preferences, rather than broader user experience or workflow integration. Similarly, the impact of the addition of these techniques to AI overlays on trust, educational or surgical outcomes remains unanswered by this study. Recognising this, these aspects have been explored in further studies, suggesting the addition of certainty estimation techniques may improve trust calibration in digital simulation (using interactive video clips) and improve operative performance in physical simulation (using high-fidelity surgical simulators)^36, 40^.

## Conclusions

This international study of surgeon perspectives on techniques to improve the interpretability of otherwise “black box” AI-assisted anatomical navigation systems showed that methods focused on conveying the certainty of AI system predictions are preferred by surgeons. In high-risk applications, such as endoscopic pituitary surgery, conveying certainty numerically or visually appears to align with surgical cognitive workflow, and demonstrates potential for fostering well-calibrated trust and appropriate reliance in these AI systems. Further studies are underway to explore the impact of these techniques on clinical performance and trust calibration.

## Supplementary Information

Supplementary Information 1.

Supplementary Information 2.

## Data Availability

Available upon reasonable request.

## References

[1] Chadebecq, F., Vasconcelos, F., Mazomenos, E. & Stoyanov, D. Computer vision in the surgical operating room. *Visc. Med.* **36**(6), 456–462. 10.1159/000511934 (2020). PMC7768144 33447601

[2] Kennedy-Metz, L. R. et al. Computer Vision in the Operating Room: Opportunities and Caveats. *IEEE Trans. Med. Robot Bionics.* **3**(1), 2–10. 10.1109/tmrb.2020.3040002 (2021). PMC7908934 33644703

[3] Varghese, C., Harrison, E. M., O’Grady, G. & Topol, E. J. Artificial intelligence in surgery. *Nat Med.* **30**(5), 1257–1268. 10.1038/s41591-024-02970-3 (2024). 38740998

[4] Mascagni, P. et al. Computer vision in surgery: From potential to clinical value. *NPJ Digit. Med.* **5**(1), 163. 10.1038/s41746-022-00707-5 (2022). PMC9616906 36307544

[5] Catchpole, K. et al. Human factors integration in robotic surgery. *Hum. Factors* **66**(3), 683–700. 10.1177/00187208211068946 (2024). PMC11268371 35253508

[6] Sarwin, G., Carretta, A., Staartjes V, Zoli M, Mazzatenta D, Regli L, et al. Anatomy Might Be All You Need: Forecasting What to Do During Surgery. *arXiv preprint arXiv:250118011*. (2025).

[7] Carstens, M. et al. The Dresden surgical anatomy dataset for abdominal organ segmentation in surgical data science. *Sci. Data* **10**(1), 3. 10.1038/s41597-022-01719-2 (2023). PMC9837071 36635312

[8] den Boer, R. B. et al. Deep learning-based recognition of key anatomical structures during robot-assisted minimally invasive esophagectomy. *Surg. Endosc.* **37**(7), 5164–5175. 10.1007/s00464-023-09990-z (2023). PMC10322962 36947221

[9] Khan, D. Z. et al. Artificial intelligence assisted operative anatomy recognition in endoscopic pituitary surgery. *NPJ Digit. Med.* **7**(1), 314. 10.1038/s41746-024-01273-8 (2024). PMC11550325 39521895

[10] Esteva, A. et al. Deep learning-enabled medical computer vision. *NPJ Digit. Med.* **4**(1), 5. 10.1038/s41746-020-00376-2 (2021). PMC7794558 33420381

[11] Fernicola, A., Palomba, G., Capuano, M., De Palma, G. D. & Aprea, G. Artificial intelligence applied to laparoscopic cholecystectomy: What is the next step? A narrative review. *Updates Surg.* **76**(5), 1655–1667. 10.1007/s13304-024-01892-6 (2024). PMC11455722 38839723

[12] Williams, S. C. et al. Artificial Intelligence Assisted Surgical Scene Recognition: A Comparative Study Amongst Healthcare Professionals. *Ann .Surg.* 10.1097/SLA.0000000000006577 (2024). PMC13056406 39474680

[13] Bhatt, U., Antorán, J., Zhang Y, Liao QV, Sattigeri P, Fogliato R, et al. Uncertainty as a form of transparency: Measuring, communicating, and using uncertainty. 401–413.

[14] Sanneman, L. & Shah, J. A. The situation awareness framework for explainable AI (SAFE-AI) and human factors considerations for XAI systems. *Int. J. Hum.-Comput. Interact.* **38**(18–20), 1772–1788 (2022).

[15] Williams, S. C. et al. A surgical approach to building impactful artificial intelligence. *NPJ Digit. Med.* **8**(1), 709. 10.1038/s41746-025-02073-4 (2025). PMC12639104 41272076

[16] Kostick-Quenet, K., Lang, B. H., Smith, J., Hurley, M. & Blumenthal-Barby, J. Trust criteria for artificial intelligence in health: Normative and epistemic considerations. *J. Med. Ethics* **50**(8), 544–551. 10.1136/jme-2023-109338 (2024). PMC11101592 37979976

[17] Amann, J., Blasimme, A., Vayena E, Frey D, Madai VI, Precise Qc. Explainability for artificial intelligence in healthcare: a multidisciplinary perspective. *BMC Med Inform Decis Mak*. 20(1):310. 10.1186/s12911-020-01332-6 (2020). PMC7706019 33256715

[18] Ud Din, S. et al. Which explainable AI methods in medical imaging are clinically impactful? A systematic literature review addressing the clinician’s perspective. *Front. Artif. Intell.* 10.3389/frai.2026.1819422 (2026) (**Systematic Review**). PMC13260647 42290694

[19] Mueller, S. T., Hoffman RR, Clancey W, Emrey A, Klein G. Explanation in human-AI systems: A literature meta-review, synopsis of key ideas and publications, and bibliography for explainable AI. *arXiv preprint arXiv:190201876*. (2019).

[20] Hoffman, R. R., Mueller ST, Klein G, Litman J. Measures for explainable ai: Explanation goodness, user satisfaction, mental models, curiosity, trust, and human-ai performance, Frontiers in Computer Science 5 (2023). https://www.frontiersin.org/journals/computer-science/articles/10.3389/fcomp.2023.1096257/full

[21] Vasey, B. et al. Reporting guideline for the early stage clinical evaluation of decision support systems driven by artificial intelligence: DECIDE-AI. *BMJ* **377**, e070904. 10.1136/bmj-2022-070904 (2022). PMC9116198 35584845

[22] Ramirez Herrera, R. et al. Optimising the usability of AI-assisted augmented reality displays of critical structures during surgery – An international study of surgeon-computer interaction. *Int. J. Hum. Comput. Stud.* 10.1016/j.ijhcs.2026.103902 (2026).

[23] Jungo, A. & Reyes, M. Assessing reliability and challenges of uncertainty estimations for medical image segmentation. In *Medical Image Computing and Computer Assisted Intervention – MICCAI 2019: 22nd International Conference, Shenzhen, China, October 13–17, 2019, Proceedings, Part II* 48–56 (Springer, 2019).

[24] Zou, K., Yuan, X., Shen X, Chen Y, Wang M, Goh RSM, et al. EvidenceCap: towards trustworthy medical image segmentation via evidential identity cap. *arXiv preprint arXiv:230100349*. (2023).

[25] Soomro, S., Niaz, A. & Choi, K. N. Grad++ScoreCAM: Enhancing visual explanations of deep convolutional networks using incremented gradient and score- weighted methods. *IEEE Access* **12**, 61104–61112. 10.1109/ACCESS.2024.3392853 (2024).

[26] Jamil, M. S., Banik, S. P., Rahaman, G. A. & Saha, S. Advanced gradcam++: Improved visual explanations of CNN decisions in diabetic retinopathy. In *Computer Vision and Image Analysis for Industry 40* 64–75 (Chapman and Hall/CRC, 2023).

[27] Borys, K. et al. Explainable AI in medical imaging: An overview for clinical practitioners–Beyond saliency-based XAI approaches. *Eur. J. Radiol.* **162**, 110786 (2023). 10.1016/j.ejrad.2023.110786 36990051

[28] Fontes, M., De Almeida, J. D. S. & Cunha, A. Application of example-based explainable artificial intelligence (XAI) for analysis and interpretation of medical imaging: A systematic review. *IEEE Access* **12**, 26419–26427 (2024).

[29] Riley, R. D. et al. Uncertainty of risk estimates from clinical prediction models: Rationale, challenges, and approaches. *BMJ*10.1136/bmj-2024-080749 (2025). PMC12128882 39947680

[30] Lambert, B., Forbes, F., Doyle, S., Dehaene, H. & Dojat, M. Trustworthy clinical AI solutions: A unified review of uncertainty quantification in deep learning models for medical image analysis. *Artif. Intell. Med.* **150**, 102830. 10.1016/j.artmed.2024.102830 (2024). 38553168

[31] Wray, C. M. & Loo, L. K. The diagnosis, prognosis, and treatment of medical uncertainty. *J. Grad. Med. Educ.* **7**(4), 523–527. 10.4300/jgme-d-14-00638.1 (2015). PMC4675405 26692960

[32] Valdenegro-Toro, M. I find your lack of uncertainty in computer vision disturbing. 1263–1272. (2021).

[33] Ali, S. et al. Explainable artificial intelligence (XAI): What we know and what is left to attain trustworthy artificial intelligence. *Inf. Fusion* **99**, 101805. 10.1016/j.inffus.2023.101805 (2023).

[34] Norgeot, B. et al. Minimum information about clinical artificial intelligence modeling: The MI-CLAIM checklist. *Nat. Med.* **26**(9), 1320–1324. 10.1038/s41591-020-1041-y (2020). PMC7538196 32908275

[35] Fogliato, R. et al. Who goes first? Influences of human-AI workflow on decision making in clinical imaging.:1362–1374 (2022).

[36] Khan, D. Z., Mao, Z., Hudson G, Wijekoon A, Chen J-e, Borg A, et al. Real-time Computer Vision Assisted Navigation for Endoscopic Pituitary Surgery: Iterative Development and Comparative Preclinical Evaluation. *medRxiv*. 26354760. 10.64898/2026.06.02.26354760 (2026).

[37] Khan DZ, Mao Z, Wijekoon A, Das A, Williams SC, Blandford A, Jain A, Harris L, Borg A, Dorward NL, Clarkson MJ, Bano S, McCulloch P, Stoyanov D, Marcus HJ. First-in-human evaluation of real-time pixel-level AIassisted anatomical segmentation in neurosurgery: pituitary surgery as an exemplar. Pituitary. 29(5):163. 10.1007/s11102-026-01762-x (2026). PMID: 42829367; PMCID: PMC13634013 PMC13634013 42829367

[38] Arun, N. et al. Assessing the trustworthiness of saliency maps for localizing abnormalities in medical imaging. *Radiol.: Artif. Intell.* **3**(6), e200267. 10.1148/ryai.2021200267 (2021). PMC8637231 34870212

[39] Khan, D. Z. et al. Video-Based Performance Analysis in Pituitary Surgery - Part 2: Artificial Intelligence Assisted Surgical Coaching. *World Neurosurg.* **190**, e79. 10.1016/j.wneu.2024.07.219 (2024). 39127380

[40] Hudson, G. R, Khan DZ, Fayez F, Bhatia S, Bano S, Costanza E, et al. Calibrating trust in AI-assisted pituitary surgery. *medRxiv* 10.64898/2026.06.02.26354735 (2026).

