# Supplementary material for: Exploring clinician interpretability of AI decision support systems for surgical anatomy recognition
Source: Sci Rep. 2026 Oct 24;16:37831. doi: 10.1038/s41598-026-74953-y (PMC13759315; doi:10.1038/s41598-026-74953-y)
Supplement: Supplementary file 2 [file 41598_2026_74953_MOESM2_ESM.docx]

# **Supplementary materials**

**Supplementary Material 1: Copy of study survey**

**Supplementary Material 2: Summary table of rank frequencies for each technique**

| **Rank** | **1^st^** | **2^nd^** | **3^rd^** | **4^th^** | **5^th^** | **6^th^** |
| --- | --- | --- | --- | --- | --- | --- |
| Certainty map | 21 | 10 | 8 | 1 | 6 | 1 |
| Certainty score | 17 | 13 | 6 | 8 | 2 | 1 |
| Scene similarity score | 1 | 13 | 8 | 12 | 10 | 3 |
| Saliency map | 3 | 8 | 12 | 9 | 8 | 7 |
| Nearest neighbours | 1 | 3 | 6 | 11 | 10 | 16 |
| No explanation | 4 | 0 | 7 | 6 | 11 | 19 |
